# Supplementary material for: Targets of the human antibody response to the variant surface glycoprotein of Trypanosoma brucei
Source: bioRxiv. 2025 Sep 25:2025.09.25.678491. Preprint. [Version 1] doi: 10.1101/2025.09.25.678491 (PMC12485828; doi:10.1101/2025.09.25.678491)
Supplement: 1 [file NIHPP2025.09.25.678491v1-supplement-1.pdf]

bioRxiv preprint doi: https://doi.org/10.1101/2025.09.25.678491; this version posted September 25, 2025. The copyright holder for this preprint (which was not certified by peer review) is the author/funder, who has granted bioRxiv a license to display the preprint in perpetuity. It is made available under aCC-BY 4.0 International license.

Supplemental Figures

Table S1: Geographical Distribution of Seroprevalent (≥ 70%) Peptides in gHAT Cohort

Peptide	Guinea	DRC	Chad
KC434873.1|225-315	9	62	4
KC434929.1|45-135	8	60	5
Tb08.27P2.630|74-163	9	55	7
Tb09.v4.0189|180-270	9	62	1
Tb427VSG-1178|45-135	8	59	6
Tb427VSG-1294|225-315	8	64	4
Tb427VSG-1320|45-135	9	59	6
Tb427VSG-1426|270-360	9	76	7
Tb427VSG-1451|180-270	9	61	5
Tb427VSG-1697|45-135	11	55	6
Tb427VSG-1710|45-135	7	57	6
Tb427VSG-2147|45-135	8	64	4
Tb427VSG-2211|0-90	10	78	7
Tb427VSG-2371|226-315	6	63	4
Tb427VSG-3213|45-135	9	64	6
Tb427VSG-538|45-135	10	54	6
Tb927.3.410|46-135	9	67	6
Tbb1125VSG-1029|45-135	6	70	1
Tbb1125VSG-1071|225-315	11	58	5
Tbb1125VSG-153|225-315	9	63	4
Tbb1125VSG-1689|90-180	7	57	6
Tbb1125VSG-246|45-135	9	64	5
Tbb1125VSG-2583|180-270	7	68	6
Tbb1125VSG-292|270-360	6	64	4
Tbb1125VSG-3176|0-90	8	62	2
Tbb1125VSG-4060|45-135	7	74	1
Tbb1125VSG-4150|135-225	9	58	3
Tbb1125VSG-454|90-180	7	60	5
Tbb1125VSG-503|45-135	4	62	6
Tbb1125VSG-5509|45-135	7	65	3
Tbb1125VSG-5577|45-135	8	59	7
Tbb1125VSG-6008|45-135	12	61	6
Tbb1125VSG-6175|0-90	6	61	3
Tbb1125VSG-6504|0-90	8	72	6
Tbb927VSG-147|45-135	9	62	5
Tbb927VSG-2130|159-249	8	58	4
Tbb927VSG-508|270-360	11	62	4
TevSTIB805.11_03.470|225-315	5	64	2
TevSTIB805.7.7120|378-468	8	66	6
**Total Patients:**	12	80	8

bioRxiv preprint doi: https://doi.org/10.1101/2025.09.25.678491; this version posted September 25, 2025. The copyright holder for this preprint (which was not certified by peer review) is the author/funder, who has granted bioRxiv a license to display the preprint in perpetuity. It is made available under aCC-BY 4.0 International license.

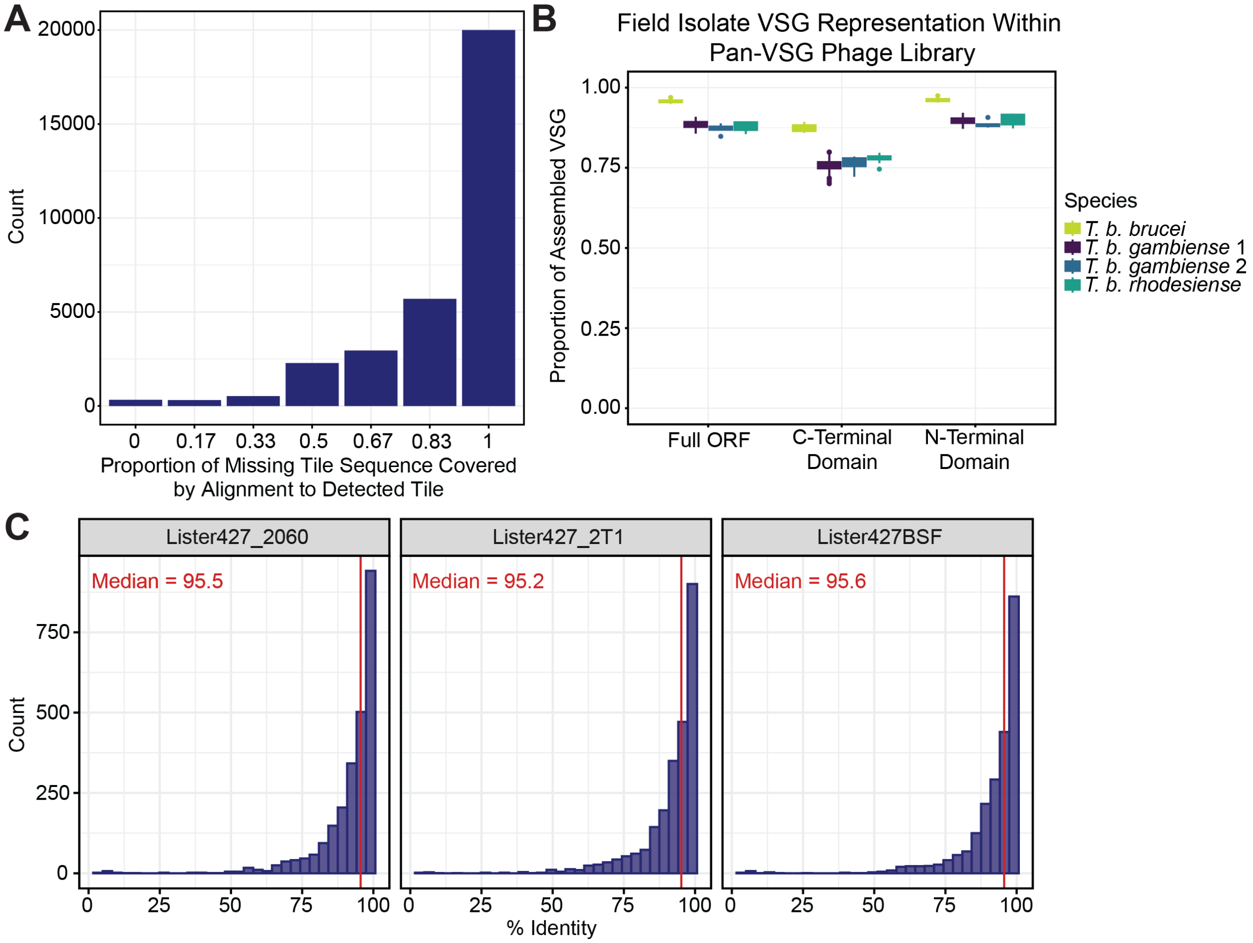

Fig. S1) Phage Library approximates VSG repertoires of uncharacterized *T. brucei* isolates. (A) Histogram showing the counts of undetected peptide tiles with homologous sequence present in represented library tiles. 28,272 of 76,601 total peptide tiles were undetected. Of the missing tiles, 11,751 contained unique sequence that was not represented by library tiles, however this rarely constituted the entire tile. **(B)** Quantification of the proportion of assembled VSG per field isolate strain where the majority (≥80%) of the whole ORF, NTD or CTD sequence can be found within Pan-VSG library tiles. **(C)** Histograms showing the distribution of alignments between assembled VSGs from three Lister427 whole genome datasets and the complete Lister427 known VSG gene reference.

bioRxiv preprint doi: https://doi.org/10.1101/2025.09.25.678491; this version posted September 25, 2025. The copyright holder for this preprint (which was not certified by peer review) is the author/funder, who has granted bioRxiv a license to display the preprint in perpetuity. It is made available under aCC-BY 4.0 International license.

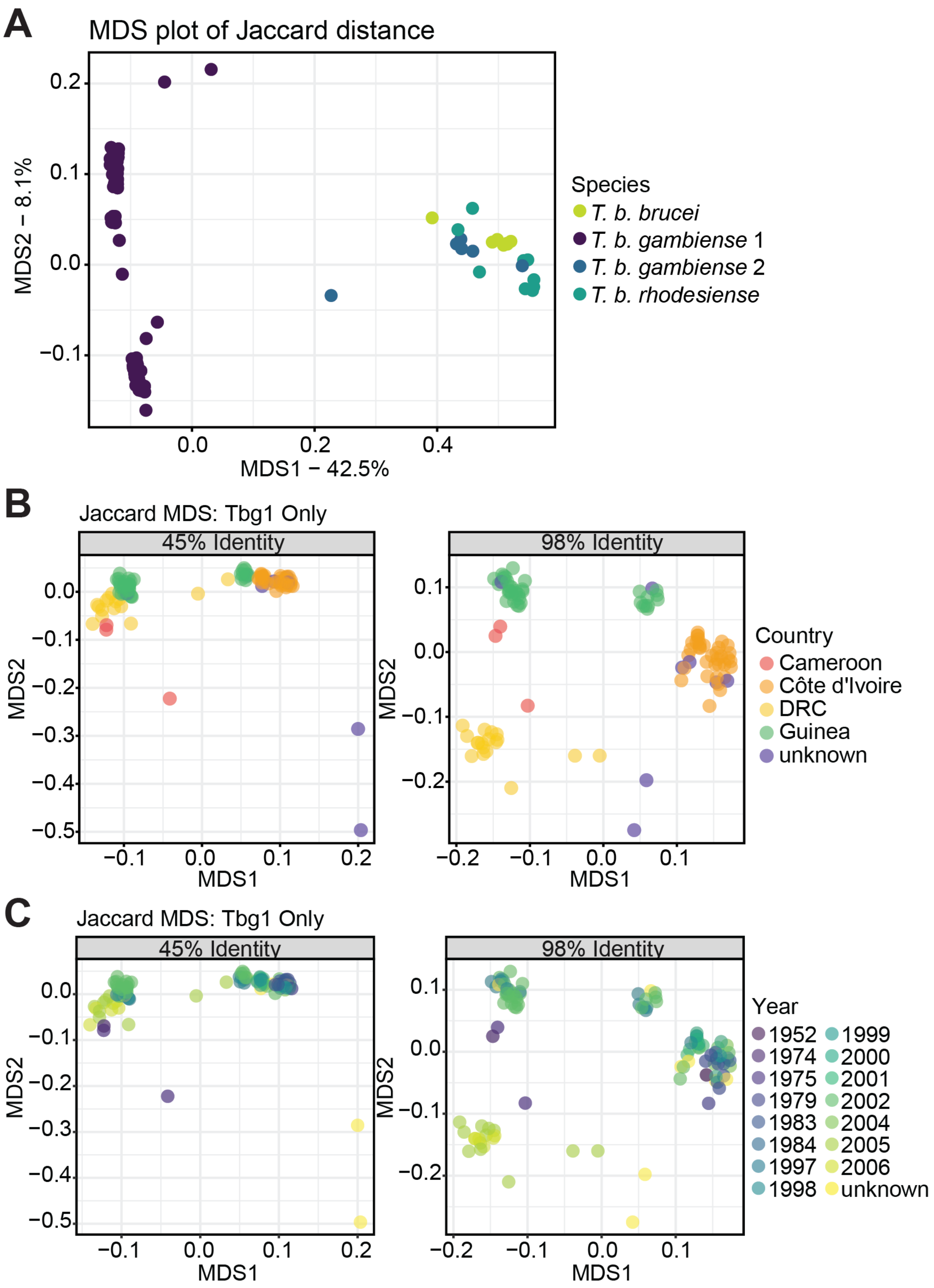

Fig. S2) *T. b. gambiense* VSG repertoires are distinct from other *T. brucei* subspecies. (A) Multidimensional scaling analysis (MDS) of the Jaccard Distance calculated based on cluster membership. Assembled VSG from all strains were clustered at a global nucleotide sequence identity of 80% using cd-hit-est. **(B-C)** MDS analysis of the Jaccard Distance

bioRxiv preprint doi: https://doi.org/10.1101/2025.09.25.678491; this version posted September 25, 2025. The copyright holder for this preprint (which was not certified by peer review) is the author/funder, who has granted bioRxiv a license to display the preprint in perpetuity. It is made available under aCC-BY 4.0 International license.

calculated based on peptide sequence clustering by cd-hit at global identity thresholds of 45% and 98%.

A

	Guinea	Democratic Republic of the Congo				Chad
	**Forecariah (N=12)**	**Dipumba (N=45)**	**Katanda (N=17)**	**Miabi (N=1)**	**Tshilenge (N=17)**	**Mobile team PNLTHA (Mandoul) (N=8)**
**Sex**						
Male	7 (58.3%)	30 (66.7%)	7 (41.2%)	0 (0%)	9 (52.9%)	7 (87.5%)
Female	5 (41.7%)	15 (33.3%)	10 (58.8%)	1 (100%)	8 (47.1%)	1 (12.5%)
**Age (years)**						
Mean (SD)	39.4 (13.7)	35.0 (12.6)	25.3 (6.44)	21.0	32.8 (11.5)	30.3 (11.1)
Median [Min, Max]	40.0 [12.0, 61.0]	35.0 [12.0, 74.0]	26.0 [12.0, 36.0]	21.0	33.0 [12.0, 48.0]	29.0 [13.0, 44.0]
**Disease Stage**						
1	12 (100%)	6 (13.3%)	6 (35.3%)	1 (100%)	17 (100%)	8 (100%)
2	0 (0%)	39 (86.7%)	11 (64.7%)	0 (0%)	0 (0%)	0 (0%)
**# Symptoms**						
Mean (SD)	4.00 (2.00)	4.71 (2.34)	5.00 (1.37)	2.00	4.59 (1.42)	3.63 (2.00)
Median [Min, Max]	4.00 [1.00, 8.00]	5.00 [0, 9.00]	5.00 [2.00, 8.00]	2.00	4.00 [2.00, 8.00]	4.00 [0, 6.00]

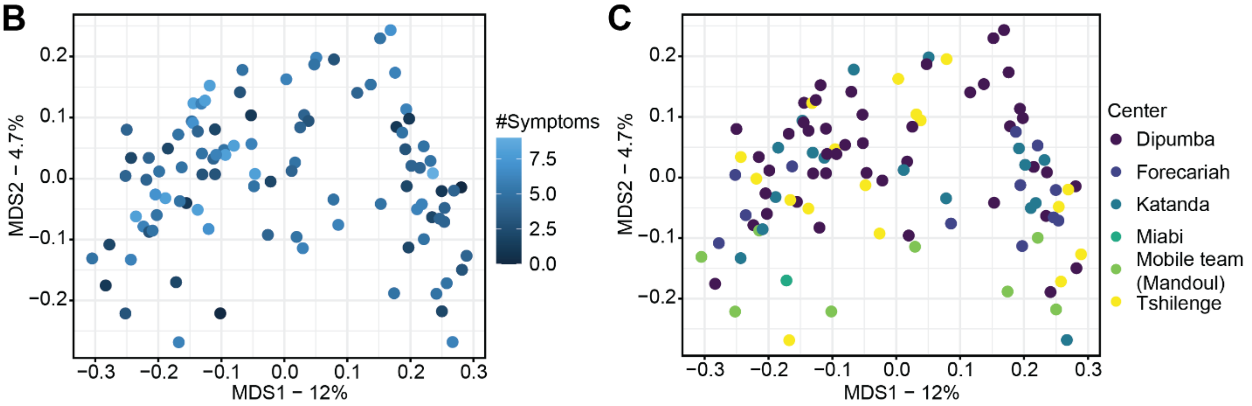

Fig. S3) Associations between symptom severity and anti-VSG response in gHAT. (A) Demographic data for the T. b. gambiense infected cohort outlining relevant metadata for subjects at each sample collection site. Limited clinical data was collected for infected persons (including fever, faintness, weight loss, swollen lymph nodes, face edema, loss of appetite, and neurological signs) which was summarized as the number of symptoms reported. Multidimensional scaling analysis of the Bray-Curtis Dissimilarity calculated based on enrichment (Log_2(FC)) of peptides in the serum of T. b. gambiense infected individuals colored by the number of recorded symptoms **(B)** and the collection site **(C)** does not reveal any strong drivers of clustering between samples.

bioRxiv preprint doi: https://doi.org/10.1101/2025.09.25.678491; this version posted September 25, 2025. The copyright holder for this preprint (which was not certified by peer review) is the author/funder, who has granted bioRxiv a license to display the preprint in perpetuity. It is made available under aCC-BY 4.0 International license.

A

	Malawi	Uganda	Tanzania
	**Rumphi (N=58)**	**Lwala (N=11)**	**Kaliua (N=1)**
**Sex**			
Male	44 (75.9%)	4 (36.4%)	1 (100%)
Female	14 (24.1%)	6 (54.5%)	0 (0%)
Missing	0 (0%)	1 (9.1%)	0 (0%)
**Age (years)**			
Mean (SD)	32.3 (12.0)	37.1 (18.6)	19.0
Median [Min, Max]	30.0 [12.0, 60.0]	34.0 [12.0, 62.0]	19.0
Missing	1 (1.7%)	1 (9.1%)	0 (0%)
**Disease Stage**			
1	9 (15.5%)	8 (72.7%)	0 (0%)
2	49 (84.5%)	3 (27.3%)	1 (100%)
**# Symptoms**			
Mean (SD)	1.09 (1.43)	4.82 (2.23)	6.00
Median [Min, Max]	0 [0, 5.00]	5.00 [2.00, 8.00]	6.00

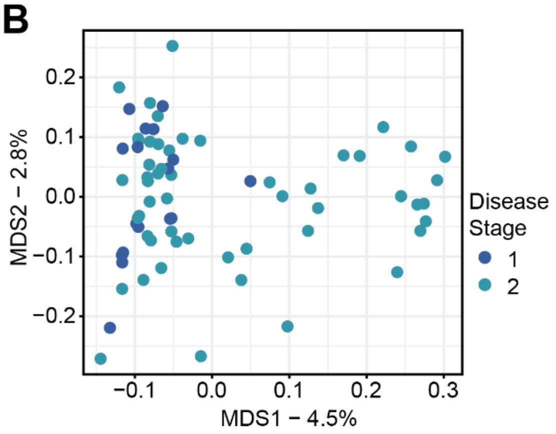

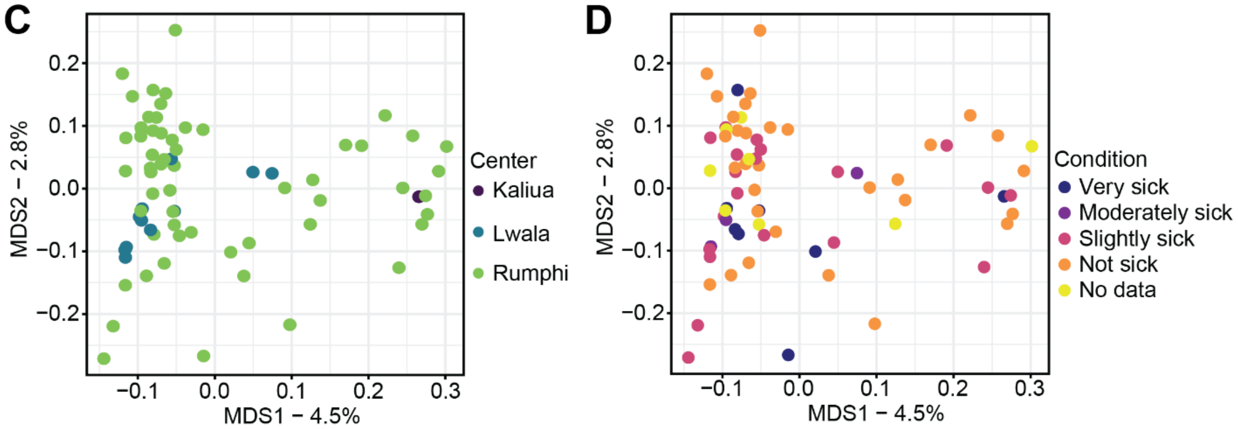

Fig. S4) Associations between symptom severity and anti-VSG response in rHAT. (A) Demographic data for the T. b. rhodesiense infected cohort outlining relevant metadata for subjects at each sample collection site. Most clinical symptoms which were recorded at other sites were not included in the Malawi Rumphi sample collection, however, qualitative assessment of physical condition was reported. **(B-D)** Multidimensional scaling analysis of the Bray-Curtis Dissimilarity calculated based on enrichment ($Log_2(FC)$) of peptides in the serum of T. b. rhodesiense infected individuals.

bioRxiv preprint doi: https://doi.org/10.1101/2025.09.25.678491; this version posted September 25, 2025. The copyright holder for this preprint (which was not certified by peer review) is the author/funder, who has granted bioRxiv a license to display the preprint in perpetuity. It is made available under aCC-BY 4.0 International license.

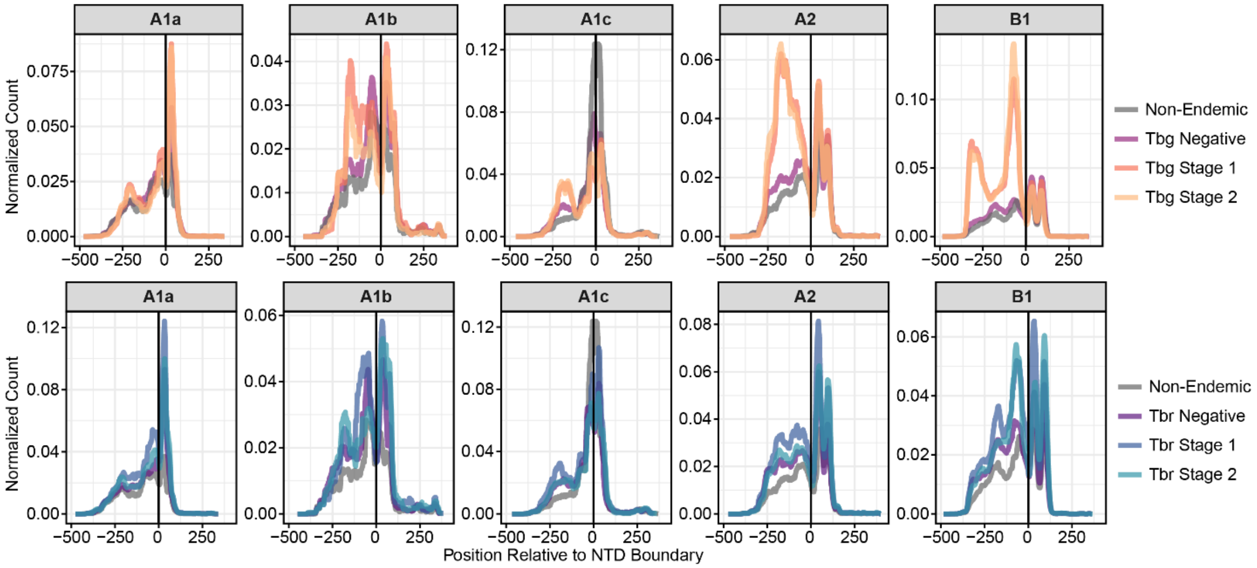

Fig. S5) Immunogenic sites along the VSG. Enriched motifs mapped to the complete repertoire of full-length VSG sequences used to create the VSG phage library. Histograms for the gHAT and rHAT cohorts showing the abundance of enriched sequences mapped to VSGs of each NTD type, normalized by cohort size and the total number of VSGs of that type. The x-axis shows the residue position along each VSG protein relative to the NTD/CTD boundary which is at x = 0. A positive x coordinate falls within the NTD and positive values are within the CTD.

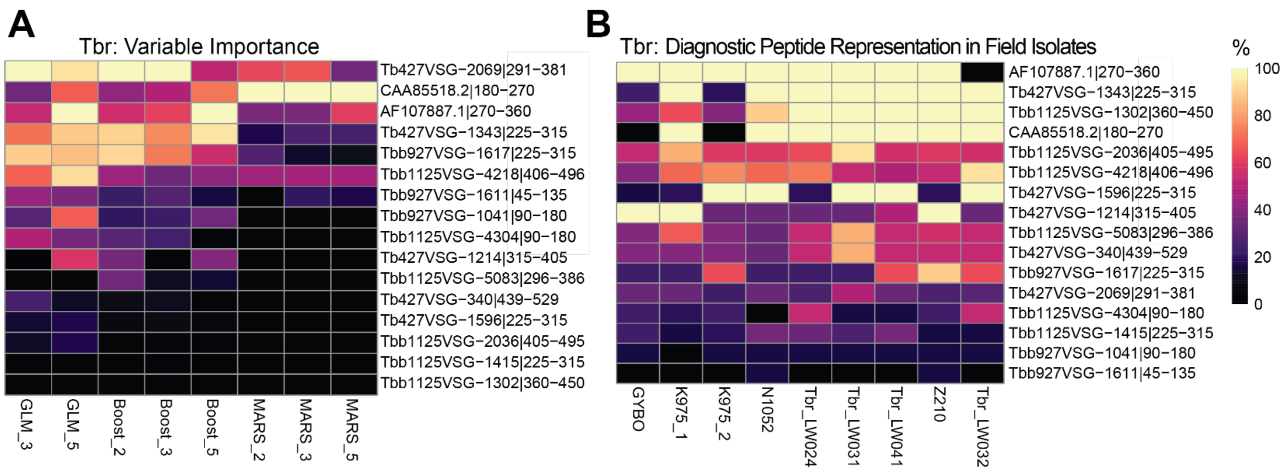

Fig. S6) Ranking rHAT diagnostic peptide candidates. Heatmaps used for the ranking of candidate peptides for the diagnosis of *T. b. rhodesiense* infection ordered by cumulative row value. Peptide identity is labeled on the y-axis. Values are normalized and displayed as either **(A)** the percentage of variable weight attributed to a peptide by each model as indicated on x-axis, or **(B)** percentage of the peptide tile sequence that could be found within VSG proteins assembled from the field isolate genomes specified on x-axis.

bioRxiv preprint doi: https://doi.org/10.1101/2025.09.25.678491; this version posted September 25, 2025. The copyright holder for this preprint (which was not certified by peer review) is the author/funder, who has granted bioRxiv a license to display the preprint in perpetuity. It is made available under aCC-BY 4.0 International license.

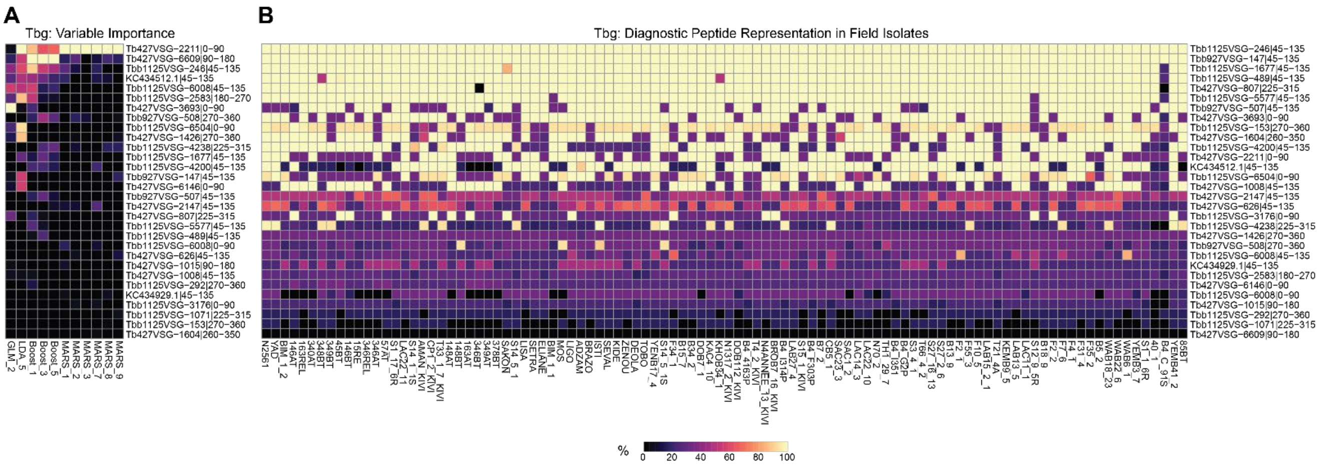

Fig. S7) Ranking gHAT diagnostic peptide candidates. Heatmaps used for the ranking of candidate peptides for the diagnosis of *T. b. gambiense* infection ordered by cumulative row value. Peptide identity is labeled on the y-axis. Values are normalized and displayed as either **(A)** the percentage of variable weight attributed to a peptide by each model as indicated on x-axis, or **(B)** percentage of the peptide tile sequence that could be found within VSG proteins assembled from the field isolate genomes specified on x-axis.
